# Prevalence, Correlates, and Description of Self-Reported Diabetes in Brazilian Capitals – Results from a Telephone Survey

**DOI:** 10.1371/journal.pone.0108044

**Published:** 2014-09-25

**Authors:** Betine Pinto Moehlecke Iser, Deborah Carvalho Malta, Bruce Bartholow Duncan, Lenildo de Moura, Álvaro Vigo, Maria Inês Schmidt

**Affiliations:** 1 Post Graduate Studies Program in Epidemiology, School of Medicine, Federal University of Rio Grande do Sul, Porto Alegre-RS, Brazil; 2 Department of Non Communicable Diseases Surveillance and Health Promotion, Secretariat for Health Surveillance, Ministry of Health, Brasilia-DF, Brazil; 3 Unit for Noncommunicable Diseases and Mental Health, Pan American Health Organization, Brasilia-DF, Brazil; Heinrich-Heine University, Faculty of Medicine, Germany

## Abstract

**Introduction:**

The prevalence of diabetes is increasing worldwide. The objective of this study is to estimate the prevalence of self-reported diabetes in Brazilian adults and to describe its population correlates as well as the clinical characteristics of the reported cases.

**Methods:**

We analyzed basic and supplementary data of 54.144 subjects participating in VIGITEL 2011 (Surveillance System for Risk and Protective Factors for Chronic Diseases), a telephone survey based on a probabilistic sample of subjects ≥18 years old residing in Brazilian state capitals and the Federal District. Estimates reported are weighted so as to represent the surveyed population.

**Results:**

The prevalence of self-reported diabetes was 6.3% (95% CI 5.9–6.7), increasing markedly with age and nutritional status, and decreasing with level of education. Prevalence was higher among those self-declaring their race/color as black. Most cases (90%) reported the diagnosis being made at 35 years or older. The vast majority (99.8%) of self-reported cases informed having previously performed at least one glucose test, and 76% of those not reporting diabetes also informed having previously performed glucose testing. Most cases (92.6%) reported following some form of diabetes treatment, 79% taking medication.

**Conclusion:**

The estimated prevalence of known diabetes found, 6.3%, is consistent with estimates given by international summaries. The additional data collected in VIGITEL 2011 regarding previous glucose testing and current treatment support the use of telephone-based information to monitor the prevalence of known diabetes in Brazilian capitals.

## Introduction

A current, worldwide epidemic of diabetes mellitus poses a challenge to health systems. The global prevalence of diabetes is estimated to increase by about 2.2% per year [1]. An estimated 46% of cases are undiagnosed [2]. The most recent estimates suggest that 11.9 million individuals between 20–79 years old currently have diabetes in Brazil [2], making it the country with the fourth largest number of diabetes cases worldwide. The prevalence of chronic disease risk factors in Brazil is estimated annually by VIGITEL (Vigilância de Fatores de Risco e Proteção para Doenças Crônicas por Inquérito Telefônico, in English, Surveillance System for Risk and Protective Factors for Chronic Diseases by Telephone Survey), which is conducted among the adult population of state capitals and the Federal District. In this survey, diabetes is defined as a self-report of a previous diabetes diagnosis by a physician [3].

Despite concerns about the validity of self-reported data [4]–[6], especially when information is collected over the phone [7], [8], the use of this methodology has grown worldwide [1], [9], due in large part to its relative speed and lower cost [10], [11], and to the difficulties of obtaining accurate information through biochemical measures in nationally representative surveys [12]. However, the quality of the information reported can be influenced by both the individual’s perception of his health status and to factors related to it [13]. Underestimation can occur as a result of only detecting cases that have already been diagnosed. Additionally, false positive reports may occur. By recognizing the prevalence of factors associated with diabetes and limitations in the use of these data, we can develop strategies to minimize these problems to extend the use of the results obtained through VIGITEL.

The aim of this study is to estimate the prevalence of self-reported diabetes in adults living in Brazilian capitals and to describe its population correlates as well as the clinical characteristics of the reported cases, based on additional questions from VIGITEL 2011 Survey.

## Methods

This is a population-based, cross-sectional study, using 2011 VIGITEL data. As data were collected through phone interviews, verbal consent was obtained from the respondent at the time of telephone contact. All interviews were recorded. VIGITEL was approved by the National Ethics Committee on Human Research of the Ministry of Health of Brazil (protocol number 355.590/2013), which waived the need for written consent.

VIGITEL uses probabilistic samples of the adult population (18 years or older) selected through listings of residential landlines in all 26 Brazilian state capitals and the Federal District. The telephone numbers used for the sample are provided without cost by the telephone companies. About 54,000 computer-assisted interviews are performed annually. For every active residential line through which contact was made with an adult resident and subsequent consent to participate in the study was obtained, a randomly selected resident was designated automatically by the system for interview.

Since 2006, the self-report of a physician diagnosis of diabetes has been used to estimate the prevalence of diabetes among the adult population [3]. In 2011, the VIGITEL questionnaire was modified. In addition to the standard question “Has a doctor ever told you that you have diabetes?” the following questions were included: “How old were you when you were diagnosed with diabetes?” (to identify the average age at diagnosis, and the duration of the disease), and “Are you currently dieting or engaging in physical activity to reduce or control your diabetes?” and “Are you currently taking any oral medicine or using insulin to control diabetes?” (to assess any measures taken for glycemic control). Additionally, questions concerning whether the participant had had his glucose level tested and if so, how long ago were asked in order to evaluate the probability of a diagnosis having been attempted.

### Statistical Methods

Prior to statistical analysis, the responses “Do not know/Did not report” were re-categorized as “No” (always less than 1% of responses, and usually less than 0.5%). The prevalence of self-reported diabetes and prevalence ratios (crude and adjusted by categories of sex, race/color, age, education, nutritional status and geographical region), in population sub-groups were calculated by means of Poisson regression with robust variance.

Data were processed and analyzed using Microsoft Excel 2010 and Stata 11.2 software. The results for categorical variables are presented as proportions with their respective 95% confidence intervals (95% CI). Average age at diagnosis and the mean duration of disease were calculated considering the respondent’s current age and reported age at time of diagnosis. For continuous variables with symmetric distributions, weighted means (standard deviations) or weighted means with 95% CIs were reported. For variables with asymmetric distributions, estimated medians and interquartile ranges (IQR) were obtained by the Kaplan-Meier method, assuming constant time.

The weights assigned to individuals interviewed were incorporated into the analyses performed in accordance with the complex sampling design used by VIGITEL, as previously described [3]. The final weight, which aimed to correct for potential biases due to differences between individuals with and without access to a landline phone, was established through a sampling design factor {1/(number of telephone lines * number of adults in the household)} and through post-stratification weighting. The latter used 48 different population strata, defined according to sex, age, and education, to expand the responses of the VIGITEL sample to the total adult population of each Brazilian capital, with weighting done according to the 2010 census of the Brazilian Institute of Geography and Statistics (IBGE) and intercensal projections [3]. To obtain a mean prevalence across all of the capitals, an additional factor which took into account the probability of drawing a telephone line within each capital was employed.

## Results

### Description of the survey sample

Between January and December of 2011, 65% of selected and dialed telephone numbers produced interviews (success rate), resulting in 54,144 adults. Only 2.2% of those contacted refused to participate. The average interview duration was 9.2 minutes. The average age of participants was 45 (16.9) years, the average number of completed years of school attended was 10.8 (4.9) years, and the average body mass index (BMI) was 25.8 (4.9) kg/m^2^.

Table 1 shows the distribution of the 54,144 participants, according to sociodemographic characteristics. The percentages were expanded so as to take into account the complex sampling design. Women predominated in the sample (53.9%), as did individuals reporting their race/color as white (44%) or brown “pardo” (41.1%). Those declaring themselves as yellow or indigenous, although less frequently present, still contributed with appreciable absolute numbers. Of note, the frequencies reported for the regions refer to capitals of that particular region. Since the number of states in each region differs and the size of the sample for each capital city was fixed, the frequencies described in the Table reflect the population of the capitals and not of the whole region.

**Table 1:** Distribution of the 54,144 participants according to sociodemographic characteristics, VIGITEL 2011.

Characteristics	N (Sample)	% Expanded^a^ (95% CI)
**Age (years)**		
18–24	6,971	16.7 (16.0–17.4)
25–34	10,147	25.4 (24.6–26.2)
35–44	10,436	20.0 (19.3–20.6)
45–54	10,359	16.6 (16.0–17.2)
55–64	8,157	11.1 (10.6–11.6)
65 and more	8,074	10.2 (9.8–10.7)
**Nutritional status** b		
Normal/Lean	24,223	50.9 (50.1–51.8)
Overweight^c^	16,982	33.3 (32.5–34.1)
Obese^d^	8,206	15.8 (15.1–16.4)
**Years of schooling**		
0–8	15,766	39.0 (38.1–39.8)
9–11	20,779	36.6 (35.9–37.4)
12 and more	17,599	24.4 (23.7–25.1)
**Sex**		
Men	21,426	46.1 (45.2–46.9)
Women	32,718	53.9 (53.1–54.8)
**Skin color** b		
White	22,990	44.0 (43.2–44.9)
Black	4,920	10.7 (10.1–11.2)
Yellow (Asian)	1,433	2.4 (2.2–2.7)
Brown	23,174	41.1 (40.3–42.0)
Indigenous (American Indian)	857	1.8 (40.3–42.0)
**Capitals in each Region**		
North	14,079	9.8 (9.5–10.1)
Northeast	18,035	25.1 (24.5–25.6)
CenterWest	8,003	11.3 (10.9–11.7)
Southeast	8,011	45.6 (44.7–46.5)
South	6,016	8.2 (7.9–8.4)

VIGITEL: Vigilância de Fatores de Risco e Proteção para Doenças Crônicas por Inquérito Telefônico (in English, Surveillance System for Risk and Protective Factors for Chronic Diseases by Telephone Survey). 95% CI: Confidence Interval of 95%.

a All analyses are weighted to represent the adult population of Brazilian capitals and the Federal District in 2011.

b The totals differ slightly from the full sample for variables which had do not know/did not reply as possible responses, as these responses were not included in the process of expanding responses in the sample to represent the total population.

c BMI 25–29.9 kg/m^2^.

d BMI≥30 kg/m^2^.

### Diabetes prevalence and correlates

**Figure 1:**
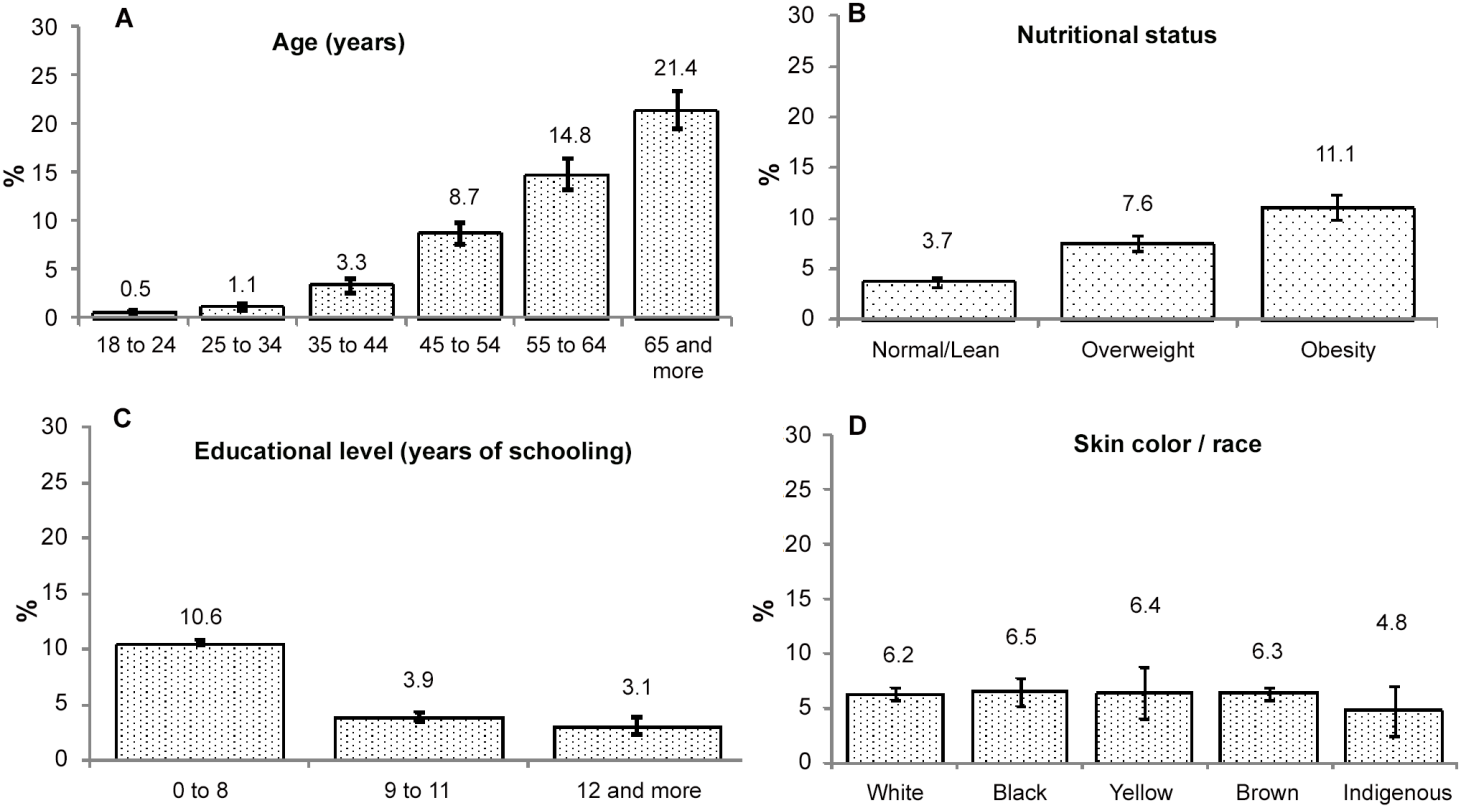
Crude prevalence of self-reported diabetes in accordance with sociodemographic factors and nutritional status. Diabetes prevalence panel: A. Prevalence according age group. B. Prevalence according nutritional status. C. Prevalence according educational level. D. Prevalence according skin color/race. Data in the combined adult population of Brazilian capital cities and the Federal District, according VIGITEL 2011. Vertical bars depict the 95% confidence limits. Percents weighted so as to represent the adult population of Brazilian capitals and the Federal District projected for the year 2011.

The frequency of a self-reported previous diagnosis of diabetes was 6.3% (95% CI: 5.9 to 6.7) for the combined population of capitals of Brazil. Figure 1 shows that the prevalence increased exponentially with age, from 0.5% in those 18–24 years old to 21.4% in those 65 or older. The prevalence also increased with increasing BMI, from 3.7% among those normoweight or lean to 11.1% among those obese. An inverse relationship was observed with level of education: the prevalence was 10.6% in those with 0–8 years of education, falling to 3.1% in those with 12 or more years of schooling. Only small differences were observed across race/color groups.

Table 2 shows prevalence and 95% CI of self-reported diabetes according to these four characteristics and according to sex and geographical region. The prevalence was slightly higher in women (6.6% versus 5.9%) and varied little across geographic regions.

**Table 2:** Prevalence of a self-reported diagnosis of diabetes mellitus and prevalence ratio according to sociodemographic factors and nutritional status in Brazilian capitals and the Federal District.

	Prevalence	Prevalence Ratio		
		Crude	Adjusted^a^	
Characteristics	% (95% CI)	PR (95% CI)	PR (95% CI)	p-value^b^
**Total**	6.3 (5.9–6.7)			
**Age (years)**				
18–24	0.5 (0.2–0.7)	1.00	1.00	
25–34	1.1 (0.7–1.5)	2.37 (1.22–4.63)	2.29 (1.11–4.73)	0.025
35–44	3.3 (2.6–4.1)	7.27 (3.96–13.37)	6.29 (3.23–12.24)	<0.001
45–54	8.7 (7.6–9.8)	18.95 (10.59–33.90)	15.52 (8.14–29.58)	<0.001
55–64	14.8 (13.2–16.5)	32.28 (18.10–57.58)	26.24 (13.78–49.96)	<0.001
65 and more	21.4 (19.5–23.3)	46.61 (26.23–82.82)	36.82 (19.23–70.48)	<0.001
**Nutritional status**				
Normal/Lean	3.7 (3.3–4.2)	1.00	1.00	
Overweight^c^	7.6 (6.7–8.4)	2.02 (1.71–2.38)	1.52 (1.29–1.78)	<0.001
Obese^d^	11.1 (9.9–12.4)	2.97 (2.52–3.51)	2.09 (1.79–2.45)	<0.001
**Years of schooling**				
0–8	10.6 (9.7–11.4)	3.40 (2.87–4.02)	1.51 (1.26–1.82)	<0.001
9–11	3.9 (3.4–4.3)	1.24 (1.03–1.50)	1.26 (1.05–1.52)	0.01
12 and more	3.1 (2.7–3.6)	1.00	1.00	
**Sex**				
Men	5.9 (5.3–6.5)	1.00	1.00	
Women	6.6 (6.1–7.1)	1.19 (0.99–1.27)	0.95 (0.83–1.08)	0.43
**Skin color/race**				
White	6.2 (5.7–6.8)	1.00	1.00	
Black	6.5 (5.1–7.8)	1.04 (0.83–1.30)	1.35 (1.07–1.70)	0.01
Yellow (Asian)	6.4 (4.1–8.7)	1.02 (0.70–1.48)	1.22 (0.81–1.83)	0.35
Brown	6.3 (5.7–6.9)	1.01 (0.89–1.16)	1.13 (0.97–1.32)	0.11
Indigenous (American Indian)	4.8 (2.4–7.1)	0.76 (0.46–1.25)	0.81 (0.50–1.30)	0.38
**Region**				
North	5.2 (4.7–5.8)	1.00	1.00	
Northeast	6.2 (5.7–6.8)	1.19 (1.04–1.36)	1.04 (0.90–1.21)	0.55
CenterWest	5.5 (4.9–6.1)	1.05 (0.90–1.23)	0.96 (0.82–1.14)	0.66
Southeast	6.8 (6.0–7.6)	1.30 (1.11–1.51)	1.05 (0.88–1.24)	0.60
South	6.0 (5.4–6.7)	1.15 (0.98–1.34)	0.92 (0.78–1.10)	0.36

VIGITEL: Vigilância de Fatores de Risco e Proteção para Doenças Crônicas por Inquérito Telefônico (in English, Surveillance System for Risk and Protective Factors for Chronic Diseases by Telephone Survey). 95% CI: Confidence Interval of 95%.

All analyses are weighted to represent the adult population of Brazilian capitals and the Federal District in 2011.

a Through Poisson regression with robust variance for all additional variables in the Table.

b Compared with the Wald statistic to the value of the reference strata.

c BMI 25–29.9 kg/m^2^.

d BMI≥30 kg/m^2^.

VIGITEL 2011.

To permit a better understanding of the differences in prevalence observed, Table 2 also provides the crude and adjusted prevalence ratios for self-reported diabetes according to the individual characteristics of respondents. After adjusting for the other characteristics presented in the Table, the principal association is with age. The next most prominent association is with nutritional status, especially obesity. Though the magnitude of the inverse association of diabetes with education was reduced with adjustment, the prevalence among those with eight or fewer years of study was 51% higher (95% CI 26% to 82%) than those with at least twelve years of schooling. Regarding race/color, after adjustment, diabetes was 35% more frequent among adults who declared their race/color as black compared to those who declared themselves as white (95% CI: 7% to 70%). Although small differences in crude prevalence were present across regions of Brazil, they disappeared after adjustment.

### Characteristics of the reported cases

**Figure 2:**
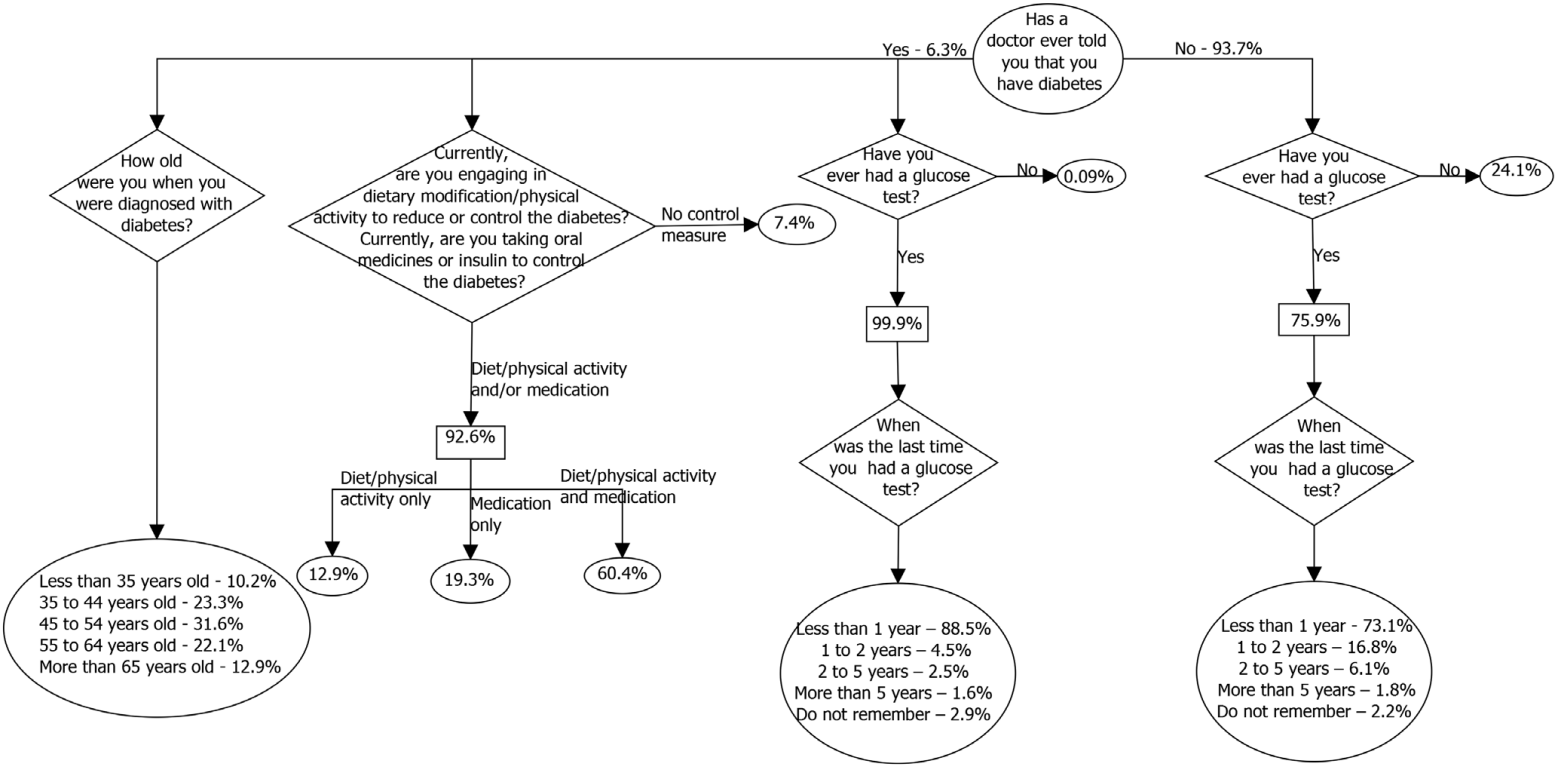
Flow diagram showing the characterization of self-reported diabetes cases. Description of the diabetes questionnaire from VIGITEL 2011: It is presented the answers according to special diabetes questionnaire applied in the 2011 version of VIGITEL. The flow starts by the circle with the basic question “Has a doctor ever told you that you have diabetes?” and must follow the results according each answer provided. The left side presents the results for those who reported having diabetes and the right side for those not reporting a previous diagnosis of diabetes. Percents weighted so as to represent the adult population of Brazilian capitals and the Federal District projected for the year 2011. VIGITEL: Vigilância de Fatores de Risco e Proteção para Doenças Crônicas por Inquérito Telefônico (in English, Surveillance System for Risk and Protective Factors for Chronic Diseases by Telephone Survey).

To characterize the study sample according to the additional questions administered in the 2011 VIGITEL survey regarding diabetes, Figure 2 illustrates the frequencies of responses obtained following the basic question “Has a doctor ever told you that you have diabetes?”.

Those who reported having diabetes were diagnosed at a mean age of 48.7 years (95% CI: 47.9 to 49.7), this being 49.3 years (95% CI: 47.8 to 50.7) for men and 48.3 years (95% CI 47.2 to 49.5) for women. For most (90%), the diagnosis occurred at or after 35 years of age. The median duration of diabetes was seven years (IQR = 11), being six years (IQR = 11) for men and seven years (IQR = 13) for women; 54% reported a disease duration of >5 years, and 19% a duration of <2 years.

Only four (0.1%) reported not having previously had a glucose test, 88.5% reported having had a glucose test within the previous year and only 4.6% reported having had their last test more than five years previously (or not remembering the date of their most recent test). Regarding treatment, 92.6% reported some type of diabetes treatment: 60.4% engaging in dietary modifications, physical activity, and drug use, and 19.3% using only oral hypoglycemic agents or insulin.

Among those not reporting a previous diagnosis of diabetes, the majority (75.9%) had undergone blood glucose testing, 73.1% of these within the last year. The likelihood of having had a blood glucose test increased with age and nutritional status (BMI categories) and demonstrated an inverse relationship with the level of education (data not shown).

For the total sample, having ever had a glucose test was reported by 77.5% (95% CI 76.7 to 78.2) of the population, this being 71.6% (95% CI: 70.4 to 72.9) for men and 82.5% (95% CI 81.7 to 83.4) for women. Of those tested, the majority (74.3%, 95% CI: 73.5 to 75.2) reported having had a glucose test within the last year.

## Discussion

The prevalence of self-reported diabetes in adults (18 year and up) residing in the capitals of Brazil in 2011 was 6.3% (95% CI: 5.9–6.7). The prevalence increased dramatically with increasing age and importantly with overweight and obesity. Lower educational level and being black were associated with greater prevalence. Only minor differences were observed between regions, which disappeared almost entirely after taking into account differences in sociodemographic factors and nutritional status.

It is important to note that these results pertain to Brazilians living in capital cities, which, according to the 2010 population census, accounts for 24% of the total Brazilian population. For comparison, the self-reported prevalence of diabetes in 2008 found in Brazilian capitals (VIGITEL) was 6.2% and in a nationally representative household survey was 5.0% [14].

Although previous studies reporting prevalence of self-reported diabetes in VIGITEL have been published [14]–[16], this is the first one to address the new set of questions added in the 2011 survey regarding glucose testing and diabetes treatment. Additionally, the prevalences here described were obtained with completely updated weighting procedures based on the results of the 2010 population census and projected for 2011. The new weights took into account recent changes in sociodemographic characteristics of population (due the increase in age and years of schooling over the period from 2000 to 2010) [17] and aimed to minimize bias resulting from incomplete landline telephone coverage.

The most recent worldwide prevalences of diabetes in adults have been estimated to be 8.3% for the year 2013 [2] and about 9.5% for the year 2008 [18]. These estimates were obtained from surveys which included a glucose measurement and, as such, are 25 to 50% higher than if only self-reported diabetes were considered [2], [19]. Thus, the 6.3% prevalence we found can be considered median to high, depending on the true proportion of undiagnosed diabetes in Brazilian capital cities.

As expected, the prevalence of diabetes in our sample increased markedly with age and with increasing BMI categories [5], [20], [21], which explains, in large part, the increase in prevalence in recent decades in the country [22] as a result of population aging and progressive increases in body weight, a trend seen worldwide [18]. The prevalence of diabetes was higher in individuals with less formal education, even after adjustment for age and nutritional status, consistent with other data from the national literature [19], [23]. The prevalence of reported diabetes among men and women was similar, this being different from a greater self-reported diabetes in women reported in the national [21], [24] and international literature [25]–[27] and generally ascribed to an increased use of health services by women [24], [28].

Since the vast majority (90%) of those who reported having diabetes were diagnosed at or after 35 years of age and the average age among both men and women was about 50 years, the cases of diabetes here described reflect predominantly type 2 diabetes [29] which generally accounts for over 90% of diagnosed cases [2], [12]. Most of those reporting diabetes had been diagnosed more than five years previously, which increases the likelihood of the condition being well established and having associated comorbidities and thus requiring greater medical care [30]. The fact that 20% of adults who reported diabetes had been diagnosed in the previous two years suggests greater recent access to diagnosis, possibly due to the expansion of the Family Health Strategy, and also the increasing incidence of diabetes [22]. This later finding is consistent with the rapid increase in the prevalence of self-reported diabetes seen temporally in VIGITEL, from 5.7% in 2006 to 7.4% in 2012 [3].

Since a diagnosis of diabetes cannot be made without performing a glycemic examination, the rare responses of a previous diagnosis of diabetes in the absence of glycemic testing were likely due to participant distraction and/or disinterest, or to a recording error, and did not materially alter the estimated prevalence of diabetes. These findings, coupled with the high frequency of glucose testing (76%) among those not reporting diabetes, support the continued use of the current VIGITEL approach as a measure of prevalence of previously known diabetes.

Adherence to drug treatment, in addition to attention to food and physical activity is fundamental for adequate glycemic control, reducing morbidity and mortality due to the disease [31]. The vast majority (92.6%) of those who reported having diabetes in our study reported engaging in some measure of treatment for the condition, such as changes in diet/physical activity levels, with 79.9% reporting the use of medication. A study also conducted in capitals of Brazil in the 1980′s found that about 20% of individuals who were aware of their diagnosis did not engage in any kind of treatment [19], suggesting improvement in this regard over the past two decades.

Potential limitations regarding the sampling process and the assumptions used to expand the data collected so as to represent the population living in capital cities merit discussion. In 2010, on average, 61% of households in capitals had a landline telephone [17]. In recent years, mobile phone ownership has increased [32], and ownership varies with years of schooling, age, race/skin color and region [33]. Although VIGITEL took into account this variation in post-stratification weighting, race/color categories were not considered in the weighting process [33], which could potentially bias results. However, a recent evaluation of the potential bias from using only landline phones conducted in two of the capitals with relatively low landline coverage showed little bias in terms of self-reported diabetes after post-stratification weighting [34], [35].

Another potential limitation is the use of self-reported, telephone-based information, as opposed to a diabetes definition based on more objective measures. Although VIGITEL has not conducted validation studies regarding its diabetes definition, other studies find reasonable sensitivities and specificities for a self-reported diagnosis of diabetes against a verified medical diagnosis [5], [30], [36]–[38], and this approach has been adopted for the planning and monitoring of preventive actions in Brazil [39]. Additionally, a population-based survey conducted in a major Brazilian city found similar prevalences of self-reported diabetes using household and phone interviews [16].

Within these limitations, our findings support the use of self-reported information for the annual monitoring of the prevalence of known diabetes in Brazilian capitals. Since the Strategic Action Plan for Confronting Chronic Non-communicable Diseases in Brazil 2011–2022 [39] defined diabetes as a priority for public health and clinical actions, the trends generated, along with other indicators, will be useful to evaluate target achievement over the next decade.

In conclusion, the estimated prevalence of known diabetes among adults ≥18 years of age in 2011 was 6.3%. The additional data collected in VIGITEL 2011, documenting frequent glucose testing in the population and treatment being undertaken in more than 90% of self-reported cases, support the use of telephone-based information to monitor the annual prevalence of known diabetes in Brazilian capitals.
